# Predictors of SARS-CoV-2 Infection in University Students: A Case-Control Study

**DOI:** 10.3390/ijerph192114376

**Published:** 2022-11-03

**Authors:** Giuseppe Migliara, Erika Renzi, Valentina Baccolini, Ambrogio Cerri, Pierluigi Donia, Azzurra Massimi, Carolina Marzuillo, Corrado De Vito, Leandro Casini, Antonella Polimeni, Eugenio Gaudio, Paolo Villari, The Collaborating Group

**Affiliations:** 1 Department of Public Health and Infectious Diseases, Sapienza University of Rome, 00185 Rome, Italy; 2 Special Office for Prevention, Protection and High Vigilance, Sapienza University of Rome, 00185 Rome, Italy; 3 Department of Oral and Maxillofacial Science, Sapienza University of Rome, 00185 Rome, Italy; 4 Department of Anatomical, Histological, Forensic Medicine and Orthopedics Sciences, Sapienza University of Rome, 00185 Rome, Italy

**Keywords:** SARS-CoV-2, high education institution, students, case-control study, risk factors

## Abstract

Closure of Higher Education Institutions in the early phase of the SARS-CoV-2 pandemic was largely diffused. With their reopening, numerous preventive measures have been enacted, but limited evidence exists on students’ behavior that could influence their infection risk. We conducted a case-control study at the Sapienza University of Rome to identify protective and risk factors for SARS-CoV-2 infection. Students attending the campus within 48 h of SARS-CoV-2 infection were considered cases. Controls were students who come in contact with a confirmed case within the campus. Demographic features and activities carried out before positivity or contact were investigated. Multivariable logistic regression models were built to identify factors associated with SARS-CoV-2 infection, estimating adjusted odds ratios (aOR) and 95% confidence intervals (95% CI). The analysis showed an increased risk of SARS-CoV-2 infection for attending the second year or above of university (aOR 17.7, 95% CI 2.21–142.82) and participating in private parties or ceremonies (aOR 15.9, 95% CI 2.30–109.67) while living outside the family (aOR 0.08, 95% CI 0.01–0.54) and attending practical activities or libraries on campus (aOR 0.29, 95% CI 0.08–0.97) reduced the risk. Data strongly suggests that it may be safe to participate in activities organized under strict infection prevention guidelines. Tailored prevention measures might reduce the risk of infection in university students.

## 1. Introduction

In response to the first wave of the SARS-CoV-2 epidemic in Italy, on 5 March 2020, the Italian government issued various restrictions on social and work activities that were deemed to incur a high risk of virus transmission [1]. Accordingly, schools at all levels and higher education institutions (HEIs) replaced face-to-face lectures with remote teaching activities. HEIs remained closed until the start of the new academic year in September 2020, when face-to-face activities partially resumed, albeit conditional on the adoption of strict prevention measures [2]. These included: a 50% reduction in classroom capacity; hybrid face-to-face/at-distance lectures; and enforcement of face masks within campus premises. The reopening of schools and universities for face-to-face activities has been debated at length, given the inherently high risk of infection in places where it is common to share closed spaces for long periods. Indeed, although in this age group infection by SARS-CoV-2 is often a- or pauci-symptomatic [3], one main concern is the possibility that a wider circulation of the virus in the student population would lead to a higher infection risk in the non-student community of cities hosting universities [4,5,6], as well as in university staff [7], who are more likely to develop severe forms of COVID-19 [8]. On the other hand, the prolonged closure of HEIs has led to a worsening of student performance [9] and of their mental health and well-being [10], suggesting that the closure of face-to-face teaching activities cannot be considered a sustainable measure in the long term. As a consequence, when they reopened in the fall of 2020, numerous universities enacted various mitigation measures to prevent campus outbreaks and to avoid further interruptions to in-person teaching activities [6,11]. Nevertheless, evidence of HEIs as enhancers of SARS-CoV-2 spread is sparse and mixed. Studies on the prevalence of SARS-CoV-2 antibodies in various university communities suggest that the circulation of the virus in these communities, before the closure of teaching activities, was essentially identical to that of the general population [12,13,14]. However, there have been reports of outbreaks on campuses after HEIs reopened [15,16,17,18], thus highlighting a possible role of these institutions in sustaining the pandemic.

To date, HEI student behavior, both on and off campus, which might affect the risk of SARS-CoV-2 infection, has not been adequately investigated. HEI activities have been guaranteed, until now, by the adoption of tight mitigation measures often accompanied by screening strategies for the early detection of SARS-CoV-2 cases [7,18,19]. However, in the current scenario, where non-pharmacological mitigation measures are being progressively attenuated or abolished altogether, it is becoming of great importance to investigate the specific occasions and types of activities involving HEI students that are most likely to increase the risk of SARS-CoV-2 transmission. Control of SARS-CoV-2 spread among students can help to bring the epidemic under control [20], especially given that the duration of protection by vaccines and their efficacy against new variants of SARS-CoV-2 is still unclear [21]. Indeed, after the first wave of the SARS-CoV-2 pandemic, when the most affected individuals were over 40 years old [22], an increase in numbers testing positive was observed in people under 25 years of age in the following months [23]. It is notable that this synchronized with the full reopening of schools and HEIs and with the emergence of the omicron variant of concern of SARS-CoV-2, B.1.1.529 [24], which is characterized by an increased capability of transmission [25] and reinfection [26].

Analyzing infection risk factors in HEI students should generate data that can adequately inform policymakers and therefore guide the elaboration of mitigation and communication measures tailored to this population, in which full compliance with non-pharmacological measures might be difficult to achieve [27] and which could be by some degree interested by the inadequate health literacy levels found in the general population [28]. This could allow infections to be kept under control in higher-education settings and therefore permit regular face-to-face teaching activities to continue for the time being without sacrificing infection control [20]. Here we present a case-control study conducted on students, not vaccinated against SARS-CoV-2, and who attended the Sapienza University of Rome between October 2020 and July 2021. We aimed to investigate how the demographic characteristics of the students, the activities they carried out within the university and the possibility of exposure to the virus outside the university might influence the risk of contracting SARS-CoV-2 infection in this population.

## 2. Materials and Methods

### 2.1. Design

This matched case-control study was conducted within the surveillance system for SARS-CoV-2 of Sapienza University of Rome and was approved by the institutional ethics board of the Umberto I teaching hospital of Rome (protocol n. 0226/2021). The surveillance system was established at the Special Office for Prevention, Protection, and High Vigilance (hereafter High Vigilance) in September 2020 to monitor and manage SARS-CoV-2 cases within the university campus. Cases among students and university staff, confirmed by antigen tests or by reverse transcription-polymerase chain reaction, were reported to High Vigilance, which then ascertained their attendance on campus premises in the 48 h preceding the onset of symptoms or the positive test. For these cases, High Vigilance would activate the contact tracing system to identify close contacts between students and university staff. Contact tracing was carried out using three tools that monitored the presence of individuals on campus: daily self-certification at the campus entrance; seat reservation in the classroom for in-person attendance of teaching activities or for study rooms; and registration by teachers of participants in face-to-face teaching activities. Moreover, cases were asked for close contacts during a telephone interview conducted with the help of the staff and the resident physicians of the Department of Public Health and Infectious Diseases. Finally, SARS-CoV-2 cases and their close contacts were notified to the relevant local health units to arrange the necessary quarantine and isolation periods. 

For this study, students who were confirmed positive for SARS-CoV-2 and had attended the campus within 48 h of the onset of symptoms or the positive test and who reported to the surveillance system of the university from October 2020 to July 2021 were considered cases. Controls were defined as students who frequented the same lectures or practical activities or the same campus premises of a confirmed case during the last day of the positive individual’s presence. Five eligible controls for each case were selected, using a random-number sequence, from students identified by contact tracing. The five eligible controls were then contacted progressively by order of selection until two students agreed to participate in the study. The random-number sequence was generate using a freely available random-number generator [29]. Both cases and controls were contacted by High Vigilance by e-mail to explain the study and to invite participants, and to ask for consent to be contacted by telephone by the staff and the resident physicians of the Department of Public Health and Infectious Diseases for the administration of a questionnaire.

### 2.2. Data Collection

The questionnaire consisted of two sections. The first section investigated the demographic and general characteristics of the case/control (gender, age, year of attendance at the university, faculty area, contacts with confirmed SARS-CoV-2 cases or with persons with symptoms of influenza-like illness (ILI), adherence to preventive measures, pre-existing medical conditions, type of housing arrangement, number of roommates, cohabitation with frail roommates, in-person working roommates and roommates under 18 years). The second section centered on activities that risked possible exposure to SARS-CoV-2, grouped into four categories comprising campus activities (involvement in practical activities or visiting libraries; consumption of food in bars, canteens, or common areas on campus), transport and travel activities (use of city public transportation, car sharing, long-range transportation, hotels), personal life activities (attendance of courses and libraries outside the university, in-person working activity, volunteer activity, use of healthcare services), and social life activities (consumption of food at bars or restaurants, visiting pubs or clubs, gyms or swimming pools, museum, cinemas or theatres, beauty salons or hairdressers, banks or post offices, shopping malls, places of worship, private parties or ceremonies, being a host or being hosted by a friend). As the study started before the onset of the vaccination campaign in Italy in December 2020, no specific question about vaccination status was included. However, 74.5% of cases included in this study were reported in October and November 2020 and vaccines initially were only available for certain categories of workers and frail subjects. Only two cases were reported in July 2021, when vaccine availability was extended to all persons over the age of 18. For cases, the investigators enquired about the possibility of exposure during the 14 days before the onset of symptoms, or during the 14 days before the positive test for asymptomatic cases. For controls, the enquiry period was the 14 days before contact with the matched case. The questionnaire was administered via telephone interviews. All 116 cases reported to High Vigilance were contacted. The final sample size included 51 cases and 102 controls matched for the date of contact.

### 2.3. Statistical Analysis

Descriptive statistics were calculated using median and interquartile range (IQR) for continuous variables and frequencies and proportions for dichotomous and categorical variables. In the first instance, conditional multivariable logistic regression models were built to estimate adjusted odds ratios (aORs) and 95% confidence intervals (CIs) for each possible exposure or general characteristic of SARS-CoV-2 infection (Model 1). Age, gender, presence of frail roommates, having pre-existing medical conditions, and contact with a known case or a person with ILI symptoms were deemed as confounders and included in each model. A second multivariate conditional regression model (Model 2) was then built including, in addition to the confounders mentioned above, all types of exposure achieving a *p*-value less than or equal to 0.35 in model 1. To avoid the ratio of events/predictors being too low [30], a best subsets variable selection was performed for model 2 with the “gvselect” Stata package [31], using the Furnival-Wilson leaps-and-bounds algorithm [32] adapted to non-normal regression models [33]. The model with the lower Akaike’s information criterion was selected [34]. All possible interaction terms between exposures were examined by adding them, one at a time, to the main effects model and were tested using a significance level cutoff of 0.05. All statistical analyses were performed using Stata (StataCorp LLC, 4905 Lakeway Drive, College Station, TX, USA) version 17.0. A two-sided *p*-value < 0.05 was considered statistically significant.

## 3. Results

### 3.1. Demographic and General Characteristics of Cases and Controls

A total of 116 confirmed cases were reported to High Vigilance from October 2020 to July 2021, of whom 51 agreed to participate in the study, achieving a response rate of 44.0%. Age and gender composition did not differ between non-responders and participants in the study. These cases were compared with 102 controls matched for date of contact, who were included after contacting 158 eligible students. Again, we found no differences in age and gender between included controls and non-responders. The general characteristics of the study population are reported in Table 1. Cases were more often female (74.5%) than controls (56.9%), while the median age was very similar between the two groups (22 years, IQR 20–24, for cases; 21.5 years, IQR 20–24 for controls). Slightly more controls than cases suffered from a pre-existing medical condition. Cases were more often enrolled in degree courses in healthcare faculties (43.1% vs. 37.3%), while the representation of first-year students was the same in both cases and controls (29.4%). With regards to housing conditions, compared to cases, controls lived less often with their family (80.4% vs. 70.6%), with underage (23.5% vs. 16.7%) and working (86.3% vs. 78.4%) roommates, and more often with frail roommates (21.6% vs. 24.5%). Adherence to face mask and hand-hygiene guidelines was very high in both groups (cases: 96.1%; controls: 94.1%), while contact with confirmed SARS-CoV-2 cases or with persons showing ILI symptoms was much higher among cases (68.6% vs. 25.5%).

### 3.2. Possibility of Exposure to SARS-CoV-2 in Cases and Controls

The activities involving both cases and controls during the enquiry period are reported in Table 2. Possibilities of exposure to SARS-CoV-2 were unevenly distributed between cases and controls. Notably, cases regularly had fewer possibilities of exposure to the virus on campus, both when undergoing practical activities and visiting libraries (41.2% vs. 51.0%), and when consuming meals (66.7% vs. 75.5%); they also used public transport more often (60.8% vs. 55.9%). In their social life, cases went to restaurants or bars (66.7% vs. 72.5%), gyms or swimming pools (13.7% vs. 17.6%), museums, cinemas, or theatres (2.0% vs. 5.9%), beauty salons or hairdressers (29.4% vs. 31.4%), banks or post offices (2.0% vs. 22.5%), shopping malls (27.5% vs. 36.3%) and places of worship (9.8% vs. 12.7%) less often than controls. Moreover, having guests at home or being hosted by friends was less common for cases (60.8% vs. 70.6%). Conversely, they frequented pubs and clubs (17.6%) and participated in private parties and ceremonies (21.6%) more often than negative students (13.7% in both categories). In their personal lives, cases attended courses and libraries outside the university (2.0% vs. 7.8%), accessed healthcare services (13.7% vs. 26.5%) and attended in-person working activities (9.8% vs. 16.7%) less frequently than controls, but participated slightly more often in volunteer activities (3.9% vs. 2.9%).

### 3.3. Predictors of Infections with SARS-CoV-2 in Cases and Controls

Results of Model 1 and Model 2 regressions are reported in Table 3. The conditional multivariate regression models adjusted for age, gender, living with frail roommates, having a pre-existing medical condition, and having had contact with a confirmed SARS-CoV-2 case or with a person with ILI symptoms (Model 1) showed a significant association between a possibility of exposure and a positive test for SARS-CoV-2 only for having participated in private parties and ceremonies (aOR 4.86, 95% CI 1.34–17.63), while visiting banks or post offices seemed to lower the risk of infection (aOR 0.09, 95% CI 0.01–0.81). No demographic or general characteristics were significantly associated with the outcome. The conditional multivariate regression model with characteristics and possibilities of exposure selected from Model 1 regressions (Model 2) indicated instead a significant increase in the risk of infection only for attending the second year or above of university (aOR 17.7, 95% CI 2.21–142.82) and participating in private parties or ceremonies (aOR 15.9, 95% CI 2.30–109.67), while not living with family members (aOR 0.08, 95% CI 0.01–0.54) and taking part in practical activities or visiting libraries on campus (aOR 0.29, 95% CI 0.08–0.97) significantly reduced the risk. No significant interaction terms between exposures were found. Among confounding variables, male gender showed a lower association with SARS-CoV-2 infection (aOR 0.20, 95% CI 0.05–0.76) while, as expected, contact with a confirmed SARS-CoV-2 case or a person with ILI symptoms greatly increased the risk (aOR 35.55, 95% CI 5.91–213.88).

## 4. Discussion

In this matched case-control study, we found that, after adjusting for other activities with the potential for exposure to the virus and potential confounders (Model 2), the risk of SARS-CoV-2 infection in the Sapienza University student community was increased only by the type of housing used by students and by their year of attendance at the university, and, among possibilities of exposure, only by attending private parties or ceremonies. No other activity seemingly increased the risk, including activities on campus.

Indeed, participating in practical activities and visiting libraries on campus premises seems to have lowered the risk of SARS-CoV-2 infection in students, in line with a previous report showing that the frequency of university courses had no effect on the risk of infection [35]. At the time of the study, numerous prevention measures were in place at Sapienza University: the capacity of classrooms was halved, use of face masks was enforced everywhere on campus, mixed face-to-face/distance learning activities were offered to students, and access to bars and canteens was limited. Moreover, a free screening test service for SARS-CoV-2 was offered to all asymptomatic students [19] and a surveillance system for SARS-CoV-2 cases in students and university staff was established by High Vigilance. Overall, these measures appear to have contributed to maintaining a safe environment on campus, probably empowered by the widespread communication campaign enacted by Sapienza University that encouraged students to focus on four basic rules when on campus, namely, hand washing, staying at home if showing symptoms, physical distancing, and mask use [36]. Interestingly, students in the second year or above appeared to be more likely to contract a SARS-CoV-2 infection. Indeed, Moro et al. reported a higher prevalence of anti-SARS-CoV-2 antibodies in postgraduate students than in undergraduates [14]. These findings may be due to the possibly wider social network of students in the higher years, together with a propensity of early-year students to adhere more closely to institution rules [37]. In any case, the higher risk in second-year students and above highlights a gap in knowledge of campus preventive measures in these students, thus pointing out the importance of correctly and continuously informing students about prevention measures to increase their compliance and consequently the effectiveness of the measures. Notably, there were no significant differences between students of healthcare and other faculties, although the former was slightly more often positive. These results contrast to some extent with the findings that healthcare students seem to have a high prevalence of previous SARS-CoV-2 contacts [14]. Indeed, while healthcare workers have a high prevalence of previous infections with SARS-CoV-2 [38] and a high risk of infection [39,40], undergraduate students probably have reduced contact with patients and access to hospital wards because of the general restrictions in access to healthcare services, thus reducing their risk of infection.

Since the beginning of the pandemic, social behavior has been recognized as a major driver of the virus spread [41]. In our study, private parties and ceremonies, such as birthdays and weddings, were associated with an increase in SARS-CoV-2 transmission risk, in line with a large body of evidence in the general population [35,40,42,43,44,45]. While public and private social activities have generally been strictly regulated or banned altogether, private parties and celebrations have more likely been able to escape control and recommended preventive measures. These possibilities of exposure may be even more important in the younger university population, given their usually wide social network, as pointed out by the very high aOR of infection for participants. Conversely, social activities often thought to be high risks, such as visiting restaurants, bars, or pubs, did not impact the risk of infection in our sample, in contrast with published data [35,40,44,46,47]. Along the same lines, other forms of social and travel activities were not associated with a higher risk of infection. Indeed, sparse and sometimes conflicting evidence exists on the risk of infection in some of these settings. Visiting gyms and receiving visitors at home have been found both to increase the risk of infection [35,43,44] and to have no effect [46,47,48]. Shopping activities [35,40,46,47,48], visits to places of worship [35,40,46], and use of public transportation [35,40,44,46,48] have more consistently been reported as representing no additional risk. Despite the discrepancies between the studies, which probably reflect the timing of their execution and heterogeneity in the preventive measures implemented in different countries, when taken together these data seem to indicate a minimal risk when visiting places open to the public, provided strict regulations are in place (access only on booking, mandatory use of face masks and social distancing, forced air ventilation, reduction in maximum capacity). Lastly, in-person working activities did not impact on infection, despite being reported as a risk factor [35,47]. This difference could underline a difference in how university students conceive and value work compared to the general population. Usually, students take paid work, often as casual labor, to be able to maintain their studies, and this could result in a more cautious attitude towards possible exposure to the virus, for fear of losing a valuable source of funds.

Household transmission has been widely recognized as a significant factor in the spread of SARS-CoV-2 [44,49,50], but while some studies reported the number of roommates [35,48] as a driver for the increased risk, we did not find this in our study. Interestingly, not living with family members appeared to have a protective role, reducing the risk of being infected. Indeed, university students who live away from their family often live with classmates or work colleagues. In this context, it is known that the household composition and the degree of intimacy has a considerable influence effect on the risk of contagion [50,51], and the number of close contacts may be lower in accommodation shared with associates, regardless of the number of roommates, compared to those that normally occur between family members. Furthermore, in our sample, only a small proportion of students resided in university accommodations, which have been suggested as places at high risk of outbreaks [15]. At odds with the literature [35,52,53], we found that living with minors of school age did not seem to have an impact on the infection risk. This might be because students, even those who live with their families, do not usually look after children at home. Moreover, at the time of the study, strict implementation of preventive measures in schools may have limited the risk of transmission via school-age children [53].

This study has several strengths and limitations. First, various biases could have affected the study. Given the often-asymptomatic course of COVID-19 in the age group targeted in the study, we could not exclude past or even current infections among the controls. Moreover, the referral of cases, although strongly recommended, was not mandatory for students. This may have led to a degree of self-selection for cases who had more interest in the issue of contact tracing and the prevention of virus spread and who were consequently more inclined to avoid behavior that represented a greater risk of transmission. Furthermore, although anonymity was granted to participants, the presence of a bias of social desirability among cases cannot be completely excluded, especially regarding the adherence to prevention measures, which was very high in both cases and controls. All these factors may ultimately have resulted in a failure to identify some associations between variables and the risk of infection. 

Second, one consequence of self-selection may have been the significant difference in the gender distribution between cases and controls, even after controlling for other confounding factors, although there is no evidence in the literature for a higher risk of infection in females. Nevertheless, in our opinion, this had little impact on the analysis, as we deem the factors investigated in the present study to be unaffected by gender. However, to take account of this imbalance, gender was always included as a confounder in all multivariable models. A stratified analysis by gender could have provided an explicit way to address an eventual residual confounding effect of this variable in the model. However, given the matched design of our study, such an approach would have been unfeasible to perform such analysis without losing too much statistical power, or giving up the matched design, which would have introduced other biases into the analysis. Moreover, the entirely randomized selection of controls from the entire population of students who came into contact with the SARS-CoV-2 positive cases assures a fair representation of the population of origin, so we are reasonably confident in the internal validity of our study. Third, as all cases and all controls attended lectures during the enquiry period, we were unable to estimate the risk for this activity, which is a major part of campus life. Nevertheless, the attendance of lectures was strictly regulated, as already mentioned, so the attributable risk should be considered negligible. Furthermore, we investigated other on-campus activities that theoretically involved a higher risk of transmission, given the potentially less strict surveillance of preventive measures compared to face-to-face lectures. Fourth, the power of the statistical analysis, given the not-very-large sample size, may not have been sufficient to highlight other significant differences between the two groups in addition to those reported. The main strength was that the case-control study design, with the recruitment of cases of SARS-CoV-2 infection and their matched controls on a given calendar day, allowed us to obtain unbiased estimates of relative risk [54]. Furthermore, although the preventive measures that were in place throughout the 10-month duration of the study and the degree of virus circulation varied extensively in Italy, matching on calendar day we were able to avoid any confounding effect of these time-dependent factors. Nevertheless, for this reason, the results of this study might not be easily generalized to other countries with significantly different prevention measures and the status of the epidemic. Finally, we were able to investigate a large number of exposure risks and possible confounders without incurring a recall bias, since the telephone interviews were carried out within a few days of reporting the case and selection of controls.

## 5. Conclusions

To the best of our knowledge, this is the first study to investigate HEI student characteristics and behavior on and off campus associated with SARS-CoV-2 infection using a case-control design. In our opinion, this study provides a meaningful advance in the debate on whether and how to fully reopen face-to-face campus activities, as our data strongly suggest that if universities and other public places are organized following strict guidelines on infection prevention measures, as was the case of Sapienza University, it may be safe to attend them even in the case of an infectious disease epidemic. Furthermore, these findings improve the knowledge of risk factors for SARS-CoV-2 infection in a population potentially at great risk of infection and of spreading the virus to elderly and frail individuals, and can therefore guide the implementation of tailored non-pharmaceutical prevention measures aimed at students.

## Figures and Tables

**Table 1:** General characteristics and contacts of SARS-CoV-2 among case and matched control students attending the Sapienza University of Rome between October 2020 and July 2021. Results are expressed as frequency (percentage) of the median (interquartile range).

		N (%)	
		Cases	Controls
**Patients**		51	102
**Gender**			
	Female	38 (74.5)	58 (56.9)
	Male	13 (25.5)	44 (43.1)
**Age, years**			
	Median (IQR)	22 (20, 24)	21.5 (20, 24)
**Faculty area**			
	Non-healthcare area	29 (56.9)	64 (62.7)
	Healthcare area	22 (43.1)	38 (37.3)
**Year of study**			
	First year	15 (29.4)	30 (29.4)
	Second and above years	36 (70.6)	72 (70.6)
**Type of housing**			
	With family	41 (80.4)	72 (70.6)
	With other roommates/university accommodation/alone	10 (19.6)	30 (29.4)
**Roommates under 18 years of age**			
	No	39 (76.5)	85 (83.3)
	Yes	12 (23.5)	17 (16.7)
**No. of roommates**			
	0 or 1	7 (13.7)	14 (13.7)
	2	16 (31.4)	29 (28.4)
	3	12 (23.5)	36 (35.3)
	4 and more	16 (31.4)	23 (22.5)
**Frail roommates**			
	No	40 (78.4)	77 (75.5)
	Yes	11 (21.6)	25 (24.5)
**In-person working roommates**			
	No	7 (13.7)	22 (21.6)
	Yes	44 (86.3)	80 (78.4)
**Coexisting condition**			
	No	43 (84.3)	80 (78.4)
	At least one	8 (15.7)	22 (21.6)
**Adherence to preventive measures (face mask and hand hygiene)**			
	Never/Rarely/Sometimes	2 (3.9)	6 (5.9)
	Often/Always	49 (96.1)	96 (94.1)
**Contact with known case or with person with ILI symptoms**			
	No	16 (31.4)	76 (74.5)
	Yes	35 (68.6)	26 (25.5)

IQR: interquartile range; ILI: influenza-like illness.

**Table 2:** Activities during the enquiry period of cases and matched-control students attending the Sapienza University of Rome between October 2020 and July 2021. Results are expressed as frequency (percentage).

	N (%)	
	Cases (n = 51)	Controls (n = 102)
**University activities**		
Attended practical activities or libraries on campus	21 (41.2)	52 (51.0)
Consumed food in bars and canteens in common areas on campus	34 (66.7)	74 (75.5)
**Transport and travel activities**		
Used public transport	31 (60.8)	54 (52.9)
Used car-sharing services	0 (0.0)	9 (8.8)
Long-range travel	11 (21.6)	22 (21.6)
Stayed in hotels	0 (0.0)	3 (2.9)
**Social life activities**		
Consumed food at bars or restaurants	34 (66.7)	74 (72.5)
Visited pubs and clubs	9 (17.6)	14 (13.7)
Visited gyms or swimming pools	9 (17.6)	19 (18.6)
Visited museums, cinemas, or theatres	1 (2.0)	6 (5.9)
Participated in private parties or ceremonies	11 (21.6)	14 (13.7)
Visited beauty salons or hairdressers	15 (29.4)	32 (31.4)
Visited banks or post offices	1 (2.0)	23 (22.5)
Visited shopping malls	14 (27.5)	37 (36.3)
Visited places of worship	5 (9.8)	13 (12.7)
Hosted (by) others outside their own family	31 (60.8)	72 (70.6)
**Personal life activities**		
Attended lessons or libraries off campus	1 (2.0)	8 (7.8)
Did volunteer activities	2 (3.9)	3 (2.9)
Carried out in-person work activity	5 (9.8)	17 (16.7)
Accessed healthcare services	7 (13.7)	27 (26.5)

**Table 3:** Risk of infection with SARS-CoV-2 in cases and matched-control students attending the Sapienza University of Rome between October 2020 and July 2021.

	Model 1 ^a^			Model 2 ^b^		
	aOR	95% CI	*p*-Value	aOR	95% CI	*p*-Value
**General characteristics**						
Faculty type (healthcare vs. non-healthcare)	0.56	0.17–1.81	0.332 ^§^			
Year of university attendance (second year and above vs. first year)	2.00	0.48–8.29	0.337 ^§^	17.7	2.21–142.82	0.007
Adherence to preventive measures (often/always vs. never/rarely/sometimes)	1.00	0.14–7.08	0.998			
Type of housing (shared apartment/campus accommodation/living alone vs. living with family)	0.36	0.11–1.16	0.087 ^§^	0.08	0.01–0.54	0.009
No. of roommates (Ref: 0 or 1)						
2	1.40	0.38–5.14	0.608			
3	0.68	0.15–2.96	0.603			
4 or more	1.70	0.41–7.07	0.465			
Roommates under 18 years of age (yes)	2.00	0.57–7.04	0.280 ^§^			
In-person working roommate (yes)	1.84	0.47–7.16	0.380			
**Campus activities**						
Attended practical training or libraries on campus (yes)	0.48	0.19–1.18	0.111 ^§^	0.29	0.08–0.97	0.045
Consumed food in bars and canteens in common areas on campus (yes)	1.24	0.49–3.15	0.652			
**Transport and travel activities**						
Used city public transportation (yes)	1.29	0.53–3.14	0.581 ^§^			
Used car-sharing services (yes)	NA	NA	NA			
Long-range travel (yes)	0.68	0.22–2.17	0.519			
Stayed in hotels (yes)	NA	NA	NA			
**Social life** **activities**						
Consumed food in bars or restaurants (yes)	0.70	0.29–1.67	0.429 ^§^			
Visited pubs or clubs (yes)	0.76	0.21–2.71	0.669			
Visited gyms or swimming pools (yes)	1.23	0.43–3.49	0.702			
Visited museums, cinemas, or theatres (yes)	0.23	0.02–2.86	0.253 ^§^			
Participated in private parties or ceremonies (yes)	4.86	1.34–17.63	0.016 ^§^	15.9	2.30–109.67	0.005
Visited beauty salons or hairdressers (yes)	1.00	0.41–2.45	0.996			
Visited banks or post offices (yes)	0.09	0.01–0.81	0.032 ^§^	0.09	0.01–1.06	0.056
Visited shopping malls (yes)	0.78	0.32–1.88	0.579			
Visited places of worship (yes)	0.68	0.20–2.34	0.544			
Hosted (by) others outside their own family (yes)	0.38	0.14–1.05	0.063 ^§^			
**Private life** **activities**						
Did volunteer work (yes)	2.30	0.27–20.2	0.452			
Carried out in-person work activity (yes)	0.48	0.12–1.92	0.301 ^§^			
Attended courses or libraries outside campus (yes)	0.22	0.02–2.66	0.234 ^§^			
Accessed healthcare services (yes)	0.52	0.16–1.68	0.275 ^§^	0.25	0.05–1.35	0.106

^a^ Conditional logistic regression adjusted for: age; gender; frail roommates, coexisting conditions, contact with confirmed SARS-CoV-2 cases, or with persons with ILI symptoms. ^b^ Conditional logistic regression adjusted for: age; gender; frail roommates, coexisting conditions, contact with confirmed SARS-CoV-2 cases or with persons with ILI symptoms, and selected exposures from models 1. ^§^ Dependent variables originally included in model 2. ILI: Influenza-like illness; aOR: adjusted odds ratio; CI: confidence interval; ref: reference.

## Data Availability

The data sets generated during and analyzed during the current study are available from the corresponding author on reasonable request.

## References

[1] (2020). Decreto Del Presidente Del Consiglio Dei Ministri Del 4 Marzo. https://www.gazzettaufficiale.it/eli/id/2020/03/04/20A01475/sg.

[2] (2020). Decreto Del Presidente Del Consiglio Dei Ministri Del 7 Agosto. https://www.gazzettaufficiale.it/eli/id/2020/08/08/20A04399/sg.

[3] Martín-Sánchez F.J., Del Toro E., Cardassay E., Valls Carbó A., Cuesta F., Vigara M., Gil P., López Picado A.L., Martínez Valero C., Miranda J.D. (2020). Clinical Presentation and Outcome across Age Categories among Patients with COVID-19 Admitted to a Spanish Emergency Department. Eur. Geriatr. Med..

[4] Viner R.M., Bonell C., Drake L., Jourdan D., Davies N., Baltag V., Jerrim J., Proimos J., Darzi A. (2021). Reopening Schools during the COVID-19 Pandemic: Governments Must Balance the Uncertainty and Risks of Reopening Schools against the Clear Harms Associated with Prolonged Closure. Arch. Dis. Child..

[5] Denny T.N., Andrews L., Bonsignori M., Cavanaugh K., Datto M.B., Deckard A., DeMarco C.T., DeNaeyer N., Epling C.A., Gurley T. (2020). Implementation of a Pooled Surveillance Testing Program for Asymptomatic SARS-CoV-2 Infections on a College Campus—Duke University, Durham, North Carolina, August 2–October 11, 2020. Morb. Mortal. Wkly. Rep..

[6] Nerhood K.J., James E.R., Hardin A., Bray J.E., Hines T.S., Young A.E., Bhavnani D. (2021). Screening Programs for SARS-CoV-2 Infections on a University Campus—Austin, Texas, September 30–November 30, 2020. Morb. Mortal. Wkly. Rep..

[7] Walke H.T., Honein M.A., Redfield R.R. (2020). Preventing and Responding to COVID-19 on College Campuses. JAMA.

[8] Gao Y.-D., Ding M., Dong X., Zhang J.-J., Kursat Azkur A., Azkur D., Gan H., Sun Y.-L., Fu W., Li W. (2021). Risk Factors for Severe and Critically Ill COVID-19 Patients: A Review. Allergy.

[9] Office for National Statistics—UK Coronavirus and Third Year or Higher Students in Higher Education, England. https://www.ons.gov.uk/peoplepopulationandcommunity/healthandsocialcare/healthandwellbeing/bulletins/coronavirusandthirdyearhighereducationstudentsengland/29novemberto20december2021.

[10] Jojoa M., Lazaro E., Garcia-Zapirain B., Gonzalez M.J., Urizar E. (2021). The Impact of COVID 19 on University Staff and Students from Iberoamerica: Online Learning and Teaching Experience. Int. J. Environ. Res. Public. Health.

[11] Blake H., Carlisle S., Fothergill L., Hassard J., Favier A., Corner J., Ball J.K., Denning C. (2022). Mixed-Methods Process Evaluation of a Residence-Based SARS-CoV-2 Testing Participation Pilot on a UK University Campus during the COVID-19 Pandemic. BMC Public Health.

[12] Tilley K., Ayvazyan V., Martinez L., Nanda N., Kawaguchi E.S., O’Gorman M., Conti D., Gauderman W.J., Van Orman S. (2020). A Cross-Sectional Study Examining the Seroprevalence of Severe Acute Respiratory Syndrome Coronavirus 2 Antibodies in a University Student Population. J. Adolesc. Health.

[13] Tuells J., Egoavil C.M., Pena Pardo M.A., Montagud A.C., Montagud E., Caballero P., Zapater P., Puig-Barberá J., Hurtado-Sanchez J.A. (2021). Seroprevalence Study and Cross-Sectional Survey on COVID-19 for a Plan to Reopen the University of Alicante (Spain). Int. J. Environ. Res. Public. Health.

[14] Muro A., Belhassen-García M., Muñoz Bellido J.L., Lorenzo Juanes H., Vicente B., Pendones J., Adserias J., Sánchez Hernández G., Rodríguez Rosa M., Vicente Villardón J.L. (2021). Seroprevalence of SARS-CoV-2 Antibodies and Factors Associated with Seropositivity at the University of Salamanca: The DIANCUSAL Study. J. Clin. Med..

[15] Wilson E., Donovan C.V., Campbell M., Chai T., Pittman K., Seña A.C., Pettifor A., Weber D.J., Mallick A., Cope A. (2020). Multiple COVID-19 Clusters on a University Campus—North Carolina, August 2020. Morb. Mortal. Wkly. Rep..

[16] Bharti N., Lambert B., Exten C., Faust C., Ferrari M., Robinson A. (2022). Large University with High COVID-19 Incidence Is Not Associated with Excess Cases in Non-Student Population. Sci. Rep..

[17] Lewis M., Sanchez R., Auerbach S., Nam D., Lanier B., Taylor J., Jaso C., Nolan K., Jacobs E.A., Hudson Parker F. (2020). COVID-19 Outbreak Among College Students After a Spring Break Trip to Mexico—Austin, Texas, March 26–April 5, 2020. Morb. Mortal. Wkly. Rep..

[18] Fox M.D., Bailey D.C., Seamon M.D., Miranda M.L. (2021). Response to a COVID-19 Outbreak on a University Campus—Indiana, August 2020. Morb. Mortal. Wkly. Rep..

[19] Baccolini V., Renzi E., Isonne C., Migliara G., Massimi A., De Vito C., Marzuillo C., Villari P. (2021). COVID-19 Vaccine Hesitancy among Italian University Students: A Cross-Sectional Survey during the First Months of the Vaccination Campaign. Vaccines.

[20] White L.F., Murray E.J., Chakravarty A. (2022). The Role of Schools in Driving SARS-CoV-2 Transmission: Not Just an Open-and-Shut Case. Cell Rep. Med..

[21] Milne G., Hames T., Scotton C., Gent N., Johnsen A., Anderson R.M., Ward T. (2021). Does Infection with or Vaccination against SARS-CoV-2 Lead to Lasting Immunity?. Lancet Respir. Med..

[22] Boehmer T.K., DeVies J., Caruso E., van Santen K.L., Tang S., Black C.L., Hartnett K.P., Kite-Powell A., Dietz S., Lozier M. (2020). Changing Age Distribution of the COVID-19 Pandemic—United States, May–August 2020. Morb. Mortal. Wkly. Rep..

[23] European Centre for Disease Prevention and Control Data on the 14-Day Age-Specific Notification Rate of New COVID-19 Cases. https://www.ecdc.europa.eu/en/publications-data/COVID-19-data-14-day-age-notification-rate-new-cases.

[24] World Health Organization Classification of Omicron (B.1.1.529): SARS-CoV-2 Variant of Concern. https://www.who.int/news/item/26-11-2021-classification-of-omicron-(b.1.1.529)-sars-cov-2-variant-of-concern.

[25] Madewell Z.J., Yang Y., Longini I.M., Halloran M.E., Dean N.E. (2022). Household Secondary Attack Rates of SARS-CoV-2 by Variant and Vaccination Status: An Updated Systematic Review and Meta-Analysis. JAMA Netw. Open.

[26] Pulliam J.R.C., van Schalkwyk C., Govender N., von Gottberg A., Cohen C., Groome M.J., Dushoff J., Mlisana K., Moultrie H. (2022). Increased Risk of SARS-CoV-2 Reinfection Associated with Emergence of Omicron in South Africa. Science.

[27] Czeisler M.É., Tynan M.A., Howard M.E., Honeycutt S., Fulmer E.B., Kidder D.P., Robbins R., Barger L.K., Facer-Childs E.R., Baldwin G. (2020). Public Attitudes, Behaviors, and Beliefs Related to COVID-19, Stay-at-Home Orders, Nonessential Business Closures, and Public Health Guidance—United States, New York City, and Los Angeles, May 5–12, 2020. Morb. Mortal. Wkly. Rep..

[28] Baccolini V., Rosso A., Di Paolo C., Isonne C., Salerno C., Migliara G., Prencipe G.P., Massimi A., Marzuillo C., De Vito C. (2021). What Is the Prevalence of Low Health Literacy in European Union Member States? A Systematic Review and Meta-Analysis. J. Gen. Intern. Med..

[29] Random Number Generator. https://www.piliapp.com/random/number/.

[30] Vittinghoff E., McCulloch C.E. (2007). Relaxing the Rule of Ten Events per Variable in Logistic and Cox Regression. Am. J. Epidemiol..

[31] Lindsey C., Sheather S. (2015). Best Subsets Variable Selection in Nonnormal Regression Models. Stata J..

[32] Furnival G.M., Wilson R.W. (1974). Regressions by Leaps and Bounds. Technometrics.

[33] Lawless J.F., Singhal K. (1978). Efficient Screening of Nonnormal Regression Models. Biometrics.

[34] Akaike H. (1974). A New Look at the Statistical Model Identification. IEEE Trans. Autom. Control.

[35] Galmiche S., Charmet T., Schaeffer L., Paireau J., Grant R., Chény O., Von Platen C., Maurizot A., Blanc C., Dinis A. (2021). Exposures Associated with SARS-CoV-2 Infection in France: A Nationwide Online Case-Control Study. Lancet Reg. Health Eur..

[36] Sapienza University of Rome Safety at Sapienza in Four Steps. https://www.uniroma1.it/en/notizia/safety-sapienza-four-steps.

[37] Jankauskiene R., Sukys S. (2012). Students’ Behaviour and Attitudes to School Rules as Outcome of Involvement in Structured Leisure Activities. Soc. Integr. Educ. Proc. Int. Sci. Conf..

[38] Paduano S., Galante P., Berselli N., Ugolotti L., Modenese A., Poggi A., Malavolti M., Turchi S., Marchesi I., Vivoli R. (2022). Seroprevalence Survey of Anti-SARS-CoV-2 Antibodies in a Population of Emilia-Romagna Region, Northern Italy. Int. J. Environ. Res. Public. Health.

[39] Rhodes S., Wilkinson J., Pearce N., Mueller W., Cherrie M., Stocking K., Gittins M., Katikireddi S.V., Tongeren M.V. (2022). Occupational Differences in SARS-CoV-2 Infection: Analysis of the UK ONS COVID-19 Infection Survey. J. Epidemiol. Community Health.

[40] Grant R., Charmet T., Schaeffer L., Galmiche S., Madec Y., Von Platen C., Chény O., Omar F., David C., Rogoff A. (2022). Impact of SARS-CoV-2 Delta Variant on Incubation, Transmission Settings and Vaccine Effectiveness: Results from a Nationwide Case-Control Study in France. Lancet Reg. Health Eur..

[41] European Centre for Disease Prevention and Control (2020). Guidelines for Non-Pharmaceutical Interventions to Reduce the Impact of COVID-19 in the EU/EEA and the UK.

[42] Whaley C.M., Cantor J., Pera M., Jena A.B. (2021). Assessing the Association Between Social Gatherings and COVID-19 Risk Using Birthdays. JAMA Intern. Med..

[43] Hobbs C.V., Martin L.M., Kim S.S., Kirmse B.M., Haynie L., McGraw S., Byers P., Taylor K.G., Patel M.M., Flannery B. (2020). Factors Associated with Positive SARS-CoV-2 Test Results in Outpatient Health Facilities and Emergency Departments Among Children and Adolescents Aged < 18 Years—Mississippi, September-November 2020. Morb. Mortal. Wkly. Rep..

[44] Arashiro T., Arima Y., Muraoka H., Sato A., Oba K., Uehara Y., Arioka H., Yanai H., Yanagisawa N., Nagura Y. (2022). Behavioral Factors Associated with SARS-CoV-2 Infection in Japan. Influenza Respir. Viruses.

[45] Thompson H.A., Mousa A., Dighe A., Fu H., Arnedo-Pena A., Barrett P., Bellido-Blasco J., Bi Q., Caputi A., Chaw L. (2021). Severe Acute Respiratory Syndrome Coronavirus 2 (SARS-CoV-2) Setting-Specific Transmission Rates: A Systematic Review and Meta-Analysis. Clin. Infect. Dis..

[46] Fisher K.A., Tenforde M.W., Feldstein L.R., Lindsell C.J., Shapiro N.I., Files D.C., Gibbs K.W., Erickson H.L., Prekker M.E., Steingrub J.S. (2020). Community and Close Contact Exposures Associated with COVID-19 Among Symptomatic Adults ≥ 18 Years in 11 Outpatient Health Care Facilities—United States, July 2020. Morb. Mortal. Wkly. Rep..

[47] Thomas D.R., Fina L.H., Adamson J.P., Sawyer C., Jones A., Nnoaham K., Barrasa A., Shankar A.G., Williams C.J. (2022). Social, Demographic and Behavioural Determinants of SARS-CoV-2 Infection: A Case-Control Study Carried out during Mass Community Testing of Asymptomatic Individuals in South Wales, December 2020. Epidemiol. Infect..

[48] Leite A., Leão T., Soares P., Severo M., Moniz M., Lucas R., Aguiar P., Meireles P., Lunet N., Nunes C. (2021). A Case-Control Study of Contextual Factors for SARS-CoV-2 Transmission. Front. Public Health.

[49] Li W., Zhang B., Lu J., Liu S., Chang Z., Peng C., Liu X., Zhang P., Ling Y., Tao K. (2020). Characteristics of Household Transmission of COVID-19. Clin. Infect. Dis. Off. Publ. Infect. Dis. Soc. Am..

[50] Madewell Z.J., Yang Y., Longini I.M., Halloran M.E., Dean N.E. (2020). Household Transmission of SARS-CoV-2: A Systematic Review and Meta-Analysis. JAMA Netw. Open.

[51] Mousa A., Winskill P., Watson O.J., Ratmann O., Monod M., Ajelli M., Diallo A., Dodd P.J., Grijalva C.G., Kiti M.C. (2021). Social Contact Patterns and Implications for Infectious Disease Transmission—A Systematic Review and Meta-Analysis of Contact Surveys. eLife.

[52] Forbes H., Morton C.E., Bacon S., McDonald H.I., Minassian C., Brown J.P., Rentsch C.T., Mathur R., Schultze A., DeVito N.J. (2021). Association between Living with Children and Outcomes from COVID-19: OpenSAFELY Cohort Study of 12 Million Adults in England. BMJ.

[53] Lessler J., Grabowski M.K., Grantz K.H., Badillo-Goicoechea E., Metcalf C.J.E., Lupton-Smith C., Azman A.S., Stuart E.A. (2021). Household COVID-19 Risk and in-Person Schooling. Science.

[54] Vandenbroucke J.P., Pearce N. (2012). Case-Control Studies: Basic Concepts. Int. J. Epidemiol..

